# Spatial calibration and PM_2.5_ mapping of low-cost air quality sensors

**DOI:** 10.1038/s41598-020-79064-w

**Published:** 2020-12-16

**Authors:** Hone-Jay Chu, Muhammad Zeeshan Ali, Yu-Chen He

**Affiliations:** National Cheng Kung University, Tainan City, Taiwan

**Keywords:** Environmental sciences, Scientific data

## Abstract

The data quality of low-cost sensors has received considerable attention and has also led to PM_2.5_ warnings. However, the calibration of low-cost sensor measurements in an environment with high relative humidity is critical. This study proposes an efficient calibration and mapping approach based on real-time spatial model. The study carried out spatial calibration, which automatically collected measurements of low-cost sensors and the regulatory stations, and investigated the spatial varying pattern of the calibrated low-cost sensor data. The low-cost PM_2.5_ sensors are spatially calibrated based on reference-grade measurements at regulatory stations. Results showed that the proposed spatial regression approach can explain the variability of the biases from the low-cost sensors with an R-square value of 0.94. The spatial calibration and mapping algorithm can improve the bias and decrease to 39% of the RMSE when compared to the nonspatial calibration model. This spatial calibration and real-time mapping approach provide a useful way for local communities and governmental agencies to adjust the consistency of the sensor network for improved air quality monitoring and assessment.

## Introduction

Air pollution is a severe global problem, especially in developing countries. A major air pollutant in urban environments is fine particulate matter (PM_2.5_), which affects human health negatively. However, the traditional existing air quality monitoring networks are sparsely deployed and lack the measurement density, because the installation and maintenance of air quality monitoring instruments are expensive. In the race to develop high-resolution, spatiotemporal air pollutant monitoring systems, low-cost air quality sensors are promising supplements to regulatory monitors for PM_2.5_ exposure assessment^1,2^. Low-cost sensors have been used to collect real-time high-density air pollution data^3,4^. Low-cost sensors for PM_2.5_ were developed from Internet of Things (IoT)^5^. Investigators can also deploy additional sensors to increase spatial coverage of air quality monitoring network^6^. Moreover, low-cost sensors can gather air quality information of the community within real time at any location. The sensors are potentially easy to use and maintain because they require less energy and space to operate^2,7^.

Low-cost sensors have been proposed to stand alone or as complementary components of existing air quality monitoring regulatory networks, which measure air pollution concentrations. However, previous studies have pointed out the inconsistency between low-cost sensors and high-quality regulatory instruments^7–9^. Low-cost observations may contain uncertainties mainly due to aerosol schemes, temperature, and high relative humidity^10,11^. The sensors may also indicate bias, as the models are dependent on location, hygroscopic growth, and specific range of relative humidity^12,13^. Each sensor type has distinct characteristics, and many factors affect light scattering, including particle size, shape, composition, and relative humidity^11^. Moreover, the particle number and mass concentrations significantly increase in high relative humidity^12^.

To minimize low-cost sensor bias and uncertainties, calibration is essential for producing validated data. Calibration should be processed before, during, and after the data collection^14^. The response of low-cost sensors could then be compared to the response of a reference instrument or a reference system. Even though low-cost sensor data attain low systematic bias after calibration, their precision is still not comparable to that of reference-grade measurements. Holstius^8^ et al. (2014) calibrated PM_2.5_ from a reference instrument with Federal Equivalent Method (FEM) status. However, keeping the regional sensors well-calibrated during deployment is a challenge^9^. Few approaches have been used to create field calibration formulas^7,15^. For example, linear models are used to adjust the raw data in certain cases^16^. Furthermore, nonlinear equations or machine learning methods are necessary to improve lower-cost sensor performance for air quality monitoring^4,15,17,18^. The reduction of measurement errors of large-scale low-cost sensor data is difficult with current multivariate calibration models^2^. To avoid regional misunderstanding of the measurements of low-cost air quality sensors, the development of a spatial calibration procedure is necessary. Thus, in the current study, we propose a real-time, regional, and simple way for low-cost sensor calibration based on reference-grade measurements at regulatory stations.

The objectives of the study are to develop the spatial varying relationship between the low-cost sensors and regulatory stations for calibration; to calibrate regional, low-cost air quality sensors against regulatory stations at one time slice; and to estimate the reliable PM_2.5_ concentration map from calibrated low-cost air quality sensors. The real-time calibration function would vary with the specific location. As a basis for the spatial calibration model, the function was assumed to be related to locations, and the coefficient estimation was carried out in spatial regression. Moreover, regional PM_2.5_ concentration estimations were conducted after the calibration. Hence, the spatial calibrated concentration from low-cost sensors would be able to identify the reliable regional pollution hotspots.

## Materials

**Figure 1 please help to update fig 1 from the attachment:**
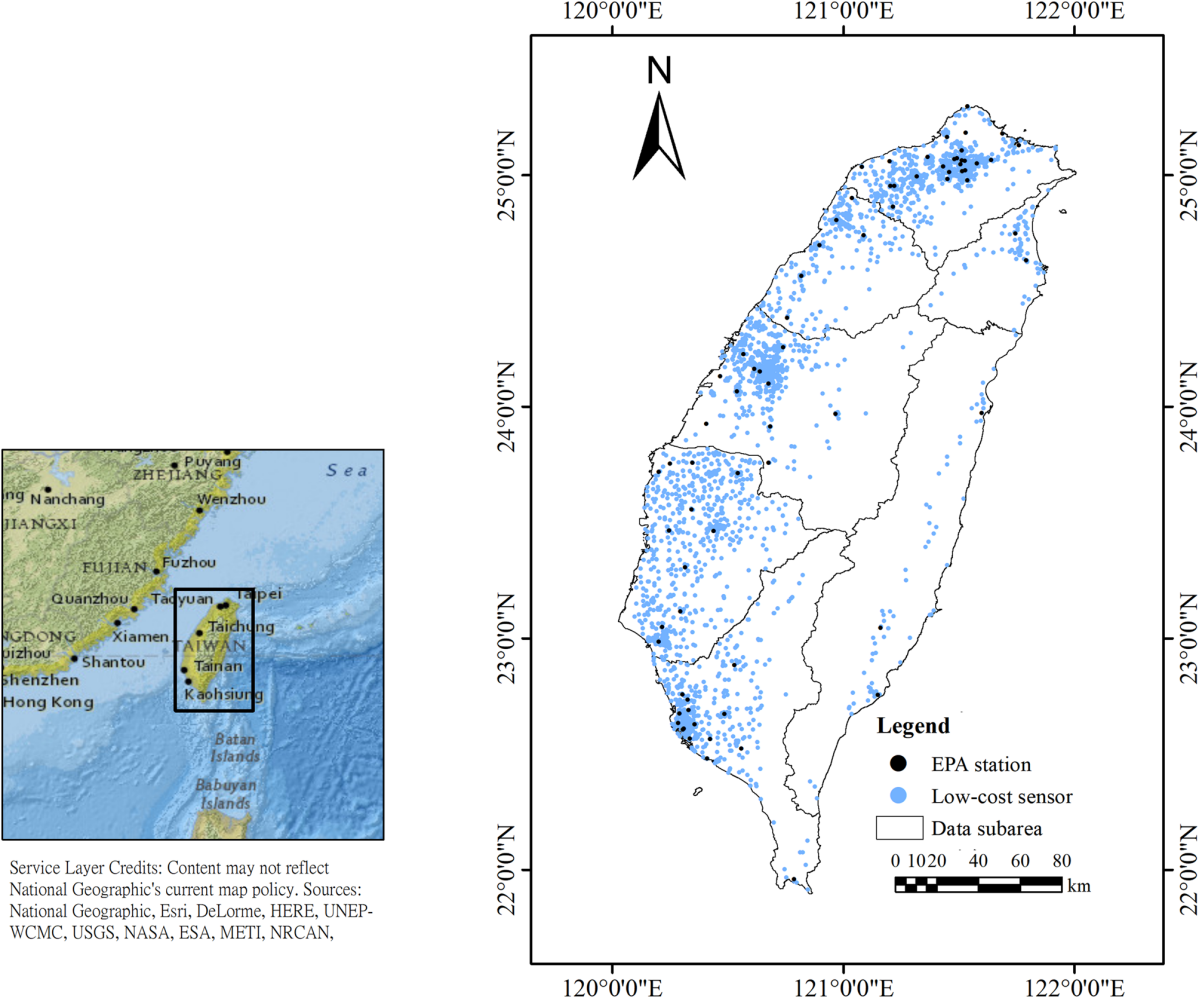
Spatial distributions of AirBox devices (green points) and TWEPA air quality monitoring stations (pink points) in Taiwan. The figure has been generated with ESRI-ArcGIS, version 10.6 (https://www.esri.com/arcgis/about-arcgis).

As the program for the low-cost PM_2.5_ sensor was launched in 2015, cooperation between the Academia Sinica and businesses, e.g., Edimax, led to the installation of thousands of boxes in Taiwan wherein local communities have volunteered to install low-cost PM_2.5_ sensors. The majority of air quality detection sensors, called AirBox, used the Realtek Ameba development board with the PMS5003 optical particulate matter sensor^5^. In this case, 2963 low-cost sensors were set-up (Fig. 1). The data are available from https://pm25.lass-net.org/. The AirBox PM_2.5_ observations along with the environmental variables, e.g., temperature (°C), and relative humidity (%), were updated roughly every 5 min.

The Taiwanese Environmental Protection Agency (TWEPA) has been regularly recording the air quality and meteorological data throughout Taiwan. Since August 2005, the TWEPA has completed the installation of 76 automatic monitoring instruments for PM_2.5_ (Fig. 1). The current study used the hourly PM_2.5_ data obtained by the TWEPA stations that complied with the regulatory air monitoring procedures. The data were accessed from https://opendata.epa.gov.tw/Data/Contents/ATM00625/.

In this case study, the PM_2.5_ hourly data obtained by the TWEPA’s Air Monitoring Network and low-cost sensor real-time data were obtained for the fine aerosol concentration in Taiwan. The time-slice at 12:00 pm (UTC), 2020-02-24 is used as a case study. At that time, the relative humidity was high, and the air quality was poor from high PM_2.5_ concentrations in the western part of Taiwan due to weak diffusion condition. The average relative humidity is 82.1% and average temperature is 22 °C in the low-cost sensors. In high relative humidity, problematic observations from the low-cost sensor are expected to be found. Temperatures between 17 and 27 °C were mostly insignificant factors of sensor bias in the study area^7^.

## Method

**Figure 2:**
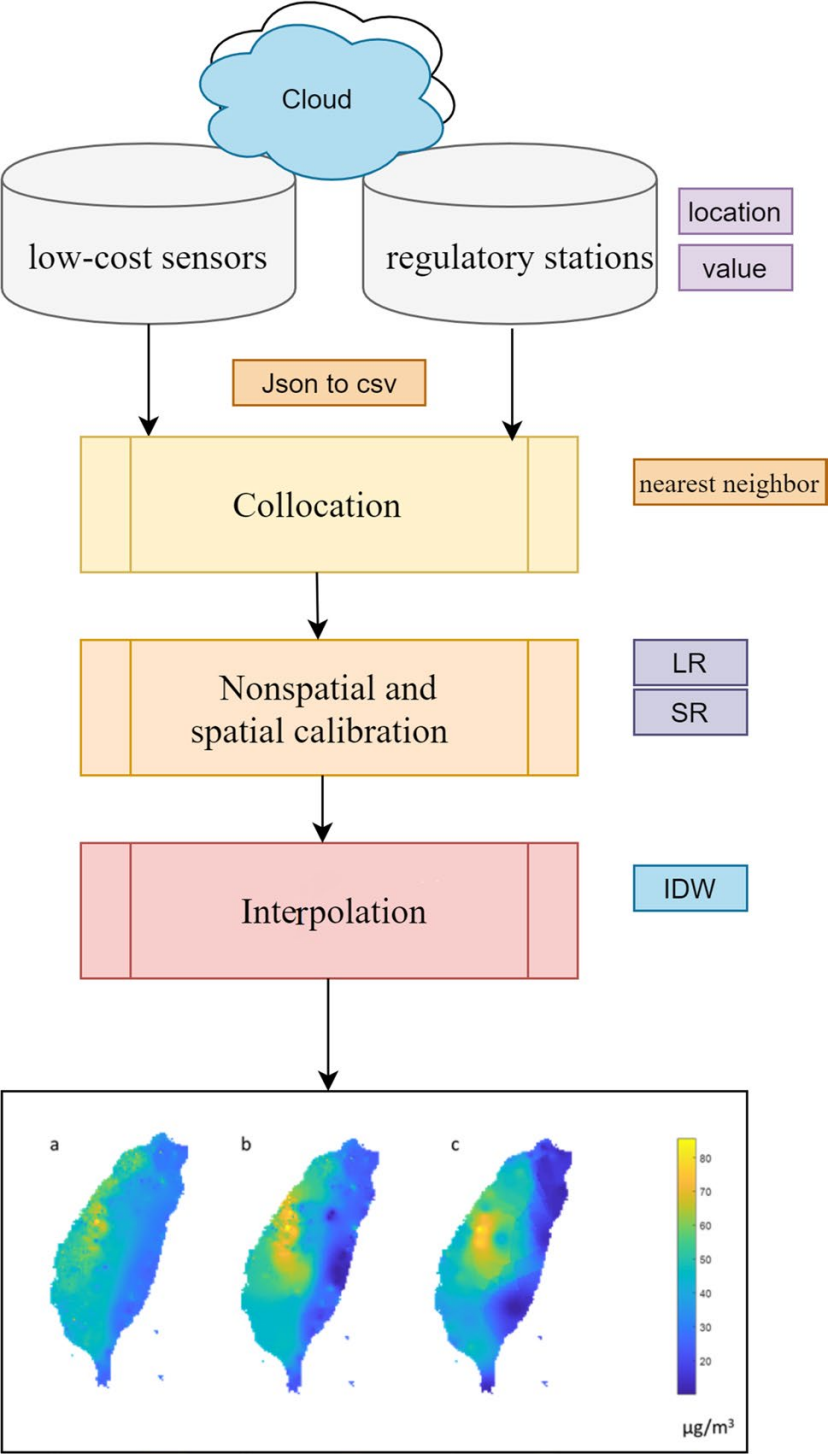
Study flowchart including data preprocessing, e.g., json to csv, data collocation, nonspatial and spatial calibration models, i.e., linear regression (LR) and spatial regression (SR), spatial interpolation, and mapping (IDW: inverse distance weighting). The bottom plot was created in MATLAB_R2018B (https://www.mathworks.com/products/matlab.html).

The main steps involved calibrating the low-cost sensors and estimating the accurate map of PM_2.5_ (Fig. 2). In the preprocessing of calibration (adjustment) model, the nearest neighbor data pair from low-cost sensor and regulatory station is used for collocation. Subsequently, the nonspatial and spatial calibration models are applied. After calibration, the regional PM_2.5_ concentrations from the sensor data are estimated.

Suppose that $n$ regulatory observations are found in the dataset for low-cost sensor calibration in the time slice. Each observation consists of a response variable ${Y}_{i}$, and $B$-dimensional covariates ${X}_{i}={\left({x}_{1i}, \dots ,{x}_{Bi}\right)}^{T}$, $i=1,\dots ,n$. Here, ${Y}_{i}$ and ${X}_{i}$ are assumed to be random variables, and $E\left({Y}_{i}|{X}_{i}\right)$ is the conditional expectation of ${Y}_{i}$ given ${X}_{i}$.

1 $$E\left({Y}_{i}|{X}_{i}\right)={\beta }_{0}+{\sum }_{b=1}^{B}{\beta }_{b}{x}_{bi}$$

Here, the calibration model considers ${x}_{1i}$, which is the PM_2.5_ concentration from the collocated low-cost sensors (nearest neighbors of regulatory observations), and ${Y}_{i}$ is the PM_2.5_ concentration of regulatory sensors from TWEPA. The low-cost sensor calibration from nonspatial calibration model (global calibration: a single coefficient set ${\beta }_{0}, { \beta }_{1}$ for all sensors) is expressed as.

2 $$E\left({Y}_{i}|{X}_{i}\right)={\beta }_{0}+{\beta }_{1}{x}_{1i}.$$

The spatial calibration model is defined from a kernel-based varying-coefficient model^19^, which is spatial regression^20^. The spatial calibration model requires the 2D coordinates of each observation as geographical information. Let $({{u}_{i},v}_{i})$ be the coordinate of the $i$th observation.

3 $$E\left({Y}_{i}|{X}_{i}, ({u}_{i},{v}_{i})\right)={\beta }_{0}\left({u}_{i},{v}_{i}\right)+{\sum }_{b=1}^{B}{\beta }_{b}\left({u}_{i},{v}_{i}\right){x}_{bi.}$$

The spatial regression coefficients, ${\beta }_{0}\left({u}_{i},{v}_{i}\right),\dots ,{\beta }_{B}\left({u}_{i},{v}_{i}\right)$, are estimated by weighted least-square method wherein the weights are evaluated by the distance between coordinates of the observed data $({u}_{i}, {v}_{i})$ and the neighborhood coordinate. Here, the PM_2.5_ of the regulatory station is the response variable and the PM_2.5_ of the collocated low-cost sensor is the covariate. Therefore, the spatial calibration model (local calibration: individual coefficient set each low-cost sensor) is expressed as.

4 $$E\left({Y}_{i}|{X}_{i}, ({u}_{i},{v}_{i})\right)={\beta }_{0}\left({u}_{i},{v}_{i}\right)+{\beta }_{1}\left({u}_{i},{v}_{i}\right){x}_{1i}.$$

In the spatial adjustment, the estimated spatial coefficient ${\widehat{\beta }}_{k}\left({u}_{i},{v}_{i}\right)$ at each observation *i* is derived from weighted least-squares

5 $${\widehat{\beta }}_{k}\left({u}_{i},{v}_{i}\right)={[{X}^{T}\mathrm{W}\left({u}_{i},{v}_{i}\right)X]}^{-1}{X}^{T}\mathrm{W}\left({u}_{i},{v}_{i}\right)Y,$$

where $\mathrm{W}\left({u}_{i},{v}_{i}\right)$ is a weight matrix based on the kernel function. The kernel distances are defined as Euclidian and Gaussian function. The Gaussian distance decay-based function is one of the typical functions of spatial autocorrelation. The geographical distance is quoted in meters. The elements in a weight matrix ${\mathrm{W}}_{ij}$ is the weighted function between observations *i* and *j*. The weight element can be computed as a kernel function

6 $${\mathrm{W}}_{ij}=exp\left(-\frac{{d}_{ij}^{2}}{{b}^{2}}\right),$$

where ${b}$ is a nonnegative parameter known as bandwidth, and ${d}_{ij}$ is the Gaussian distance decay-based function based on the geographical distance. The optimal bandwidth is determined by the criteria, i.e., cross validation. Meanwhile, the model residual (${e}_{i}$) at observation *i* is defined as

7 $${e}_{i}={Y}_{i}-{Y}_{i}^{^{\prime}},$$

where ${Y}_{i}$ is the observed concentration from regulatory station *i*, and ${Y}_{i}^{^{\prime}}$ is the calibrated concentration of the low-cost sensors near regulatory station *i*. To evaluate the model performance, the common measures, R^2^ and RMSEs, are used based on the observation data and the estimated values at the regulatory stations.

Furthermore, the inverse distance weighting (IDW) method is a straightforward and low-computational approach for spatial interpolation procedures of the PM_2.5_ concentration map. The IDW method is used in this study to generate a 2 km-resolution PM_2.5_ concentration map after spatial calibration. The weights of the observations in the IDW are based on the inverse distances of the unknown point and observations. The parameter power value is 2 as the inverse distance squared weighted interpolation.

## Results

### Data description

**Figure 3:**
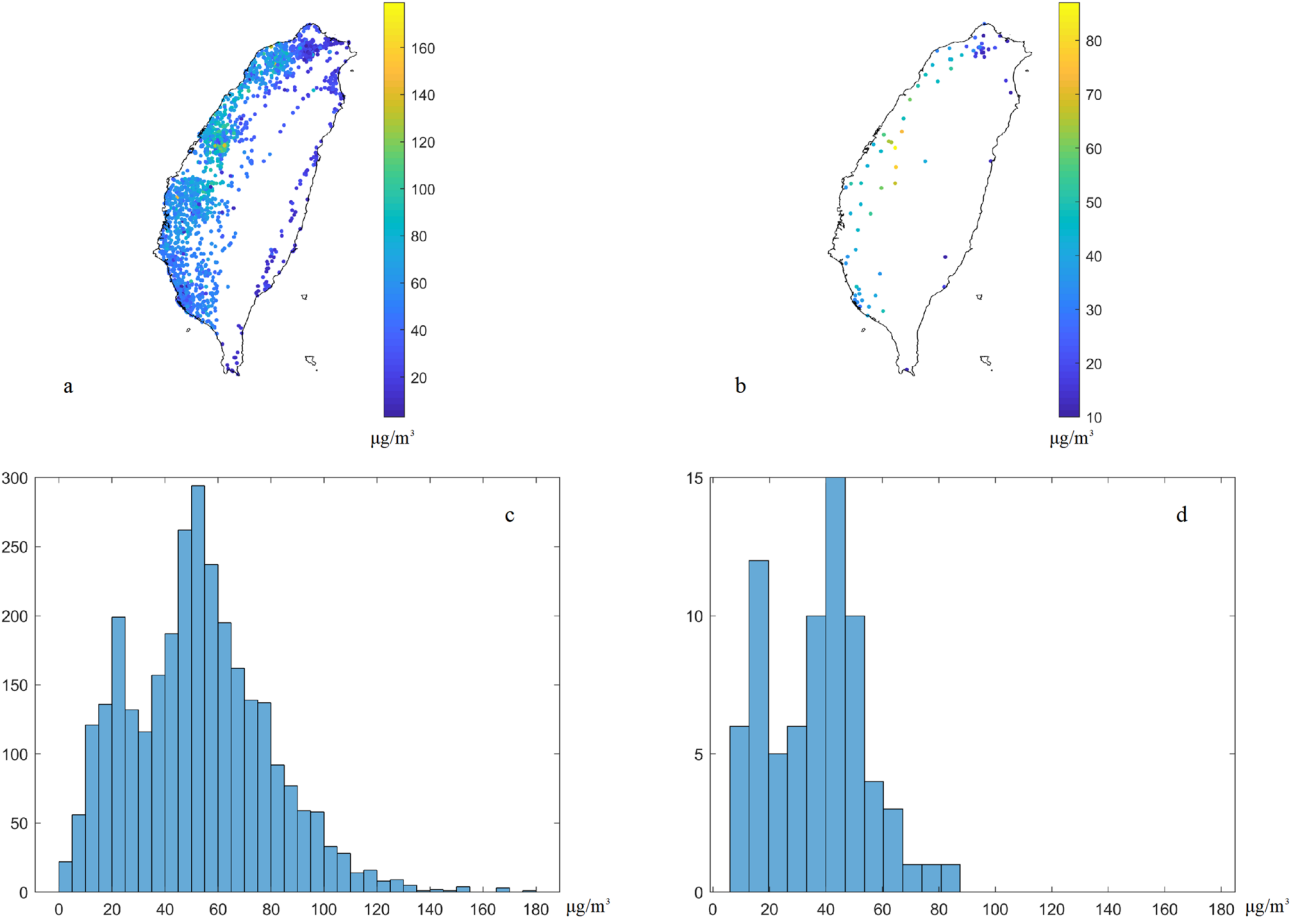
Raw PM_2.5_ concentrations from (**a**) low-cost sensors and (**b**) regulatory stations and their histograms (**c**, **d**) in the case study. The plots were created in MATLAB_R2018B (https://www.mathworks.com/products/matlab.html).

The raw PM_2.5_ concentrations from (a) low-cost sensor and (b) regulatory stations and their corresponding histograms (c and d) are shown in Fig. 3. The peaks of raw low-cost sensor data distribution are approximately over 20 and 50–60 μg/m^3^, but the peaks of raw regulatory station data are 10–20, 40–50 μg/m^3^, respectively. In addition, the average values of low-cost sensors and regulatory sensors are 51.9 and 36.7 μg/m^3^, and the standard derivations are 26.1 and 17.5 μg/m^3^, respectively. The average values of the low-cost sensor data are double than those of the regulatory sensors. Furthermore, the variance of low-cost sensor data is significantly larger than the EPA monitoring data (681 and 306 (μg/m^3^)^2^ in the low-cost sensor and EPA data, respectively). The low-cost sensor data calibration is needed in the condition. Most PM_2.5_ measurements from raw low-cost sensors and regulatory stations showed extremely high correlation with the time series, and these can be used to identify the PM_2.5_ pollution hotspots^7^. However, the correlation of low-cost sensors and regulatory sensor data is only 0.8 from the collocated stations in this time slice.

### Spatial and nonspatial calibration models

**Figure 4:**
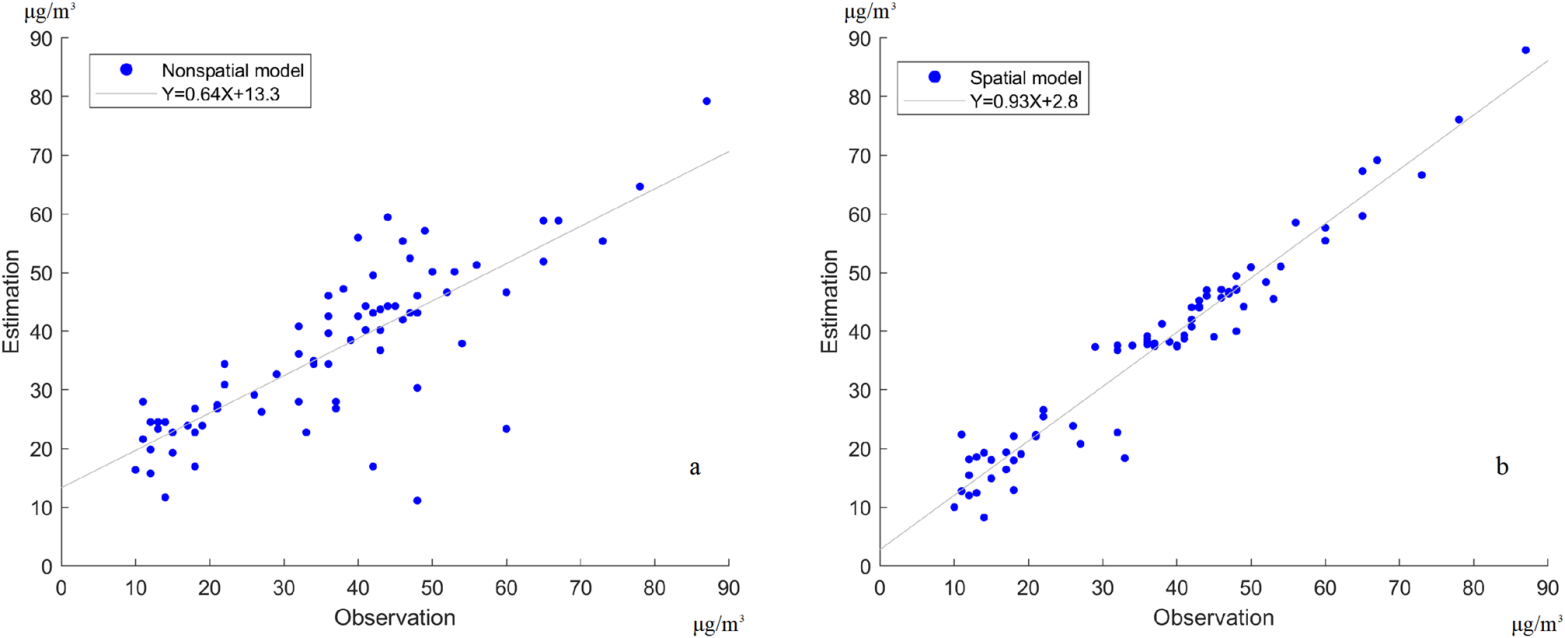
Calibrated results (estimations) when compared to observations in the (**a**) nonspatial and (**b**) spatial calibration models (slope: 0.64 and 0.93; intercept: 13.3 and 2.8 in the nonspatial and spatial calibrations, respectively).

**Figure 5:**
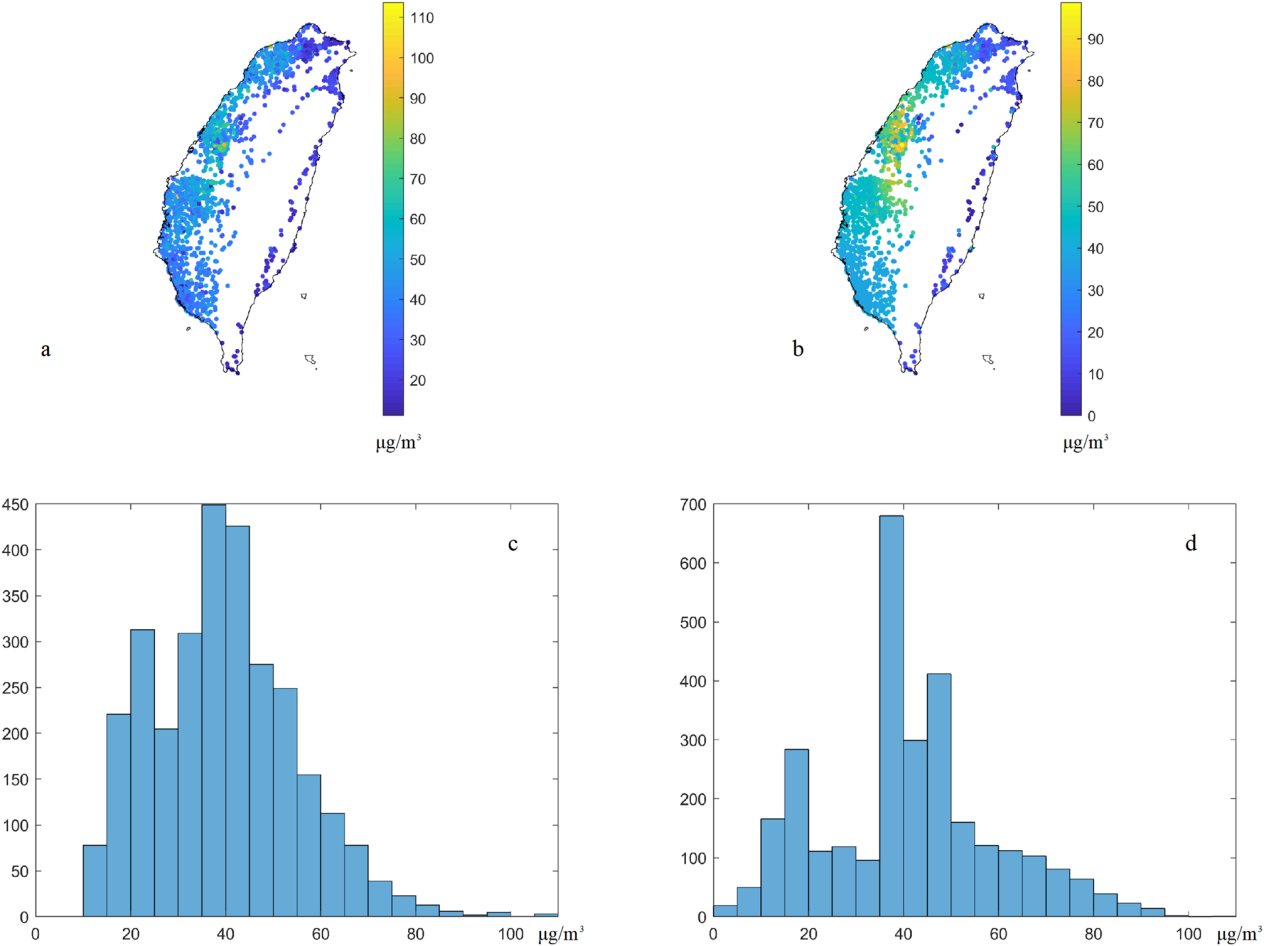
Spatial patterns of calibrated PM_2.5_ based on the (**a**) nonspatial and (**b**) spatial calibration models, and the data distribution of calibrated PM_2.5_ from the (**c**) nonspatial and (**d**) spatial calibration models. The plots were created in MATLAB_R2018B (https://www.mathworks.com/products/matlab.html).

The nonspatial and spatial calibration models have R-square values of 0.64 and 0.94, respectively (Fig. 4). The RMSE of the low-cost sensor is modified from 17.7 to 10.5 μg/m^3^ after calibration from the nonspatial model, but can be improved to 4.1 μg/m^3^ using the spatial calibration model. Considering the spatial calibration model, the model performance improved, that is, the spatial scheme can reduce the inconsistency of low-cost sensors and the neighboring regulatory observations. Figure 5 shows the spatial pattern and data distribution of calibrated results based on nonspatial and spatial calibration models. The data distribution considering the nonspatial model is similar to the original low-cost sensor data. After calibrating by spatial model, the peaks of data distribution are 10–20, and 40–50 μg/m^3^, respectively, and the standard derivation decreased from 26.1 to 18.0 μg/m^3^. The distribution of calibrated data in the spatial model is transformed to similar data distribution of regulatory stations. The result shows that the spatial calibration model performed better than the nonspatial calibration model.

The spatial calibration model is a local linear transformation used to model spatially varying relationships^20^. However, the nonspatial approach, i.e., global linear transformation, can lead to biased estimations of regression model parameters due to the sample outliers. The proposed spatial calibration model is based on a weighted local fitting approximation to the function being estimated. The approach adopted in this study can identify a nonlinear and spatial varying relation between a pair of low-cost sensors and regulatory stations. The spatial calibrated data can be used directly to estimate a reliable air quality map.

### Spatial calibration coefficient

**Figure 6:**
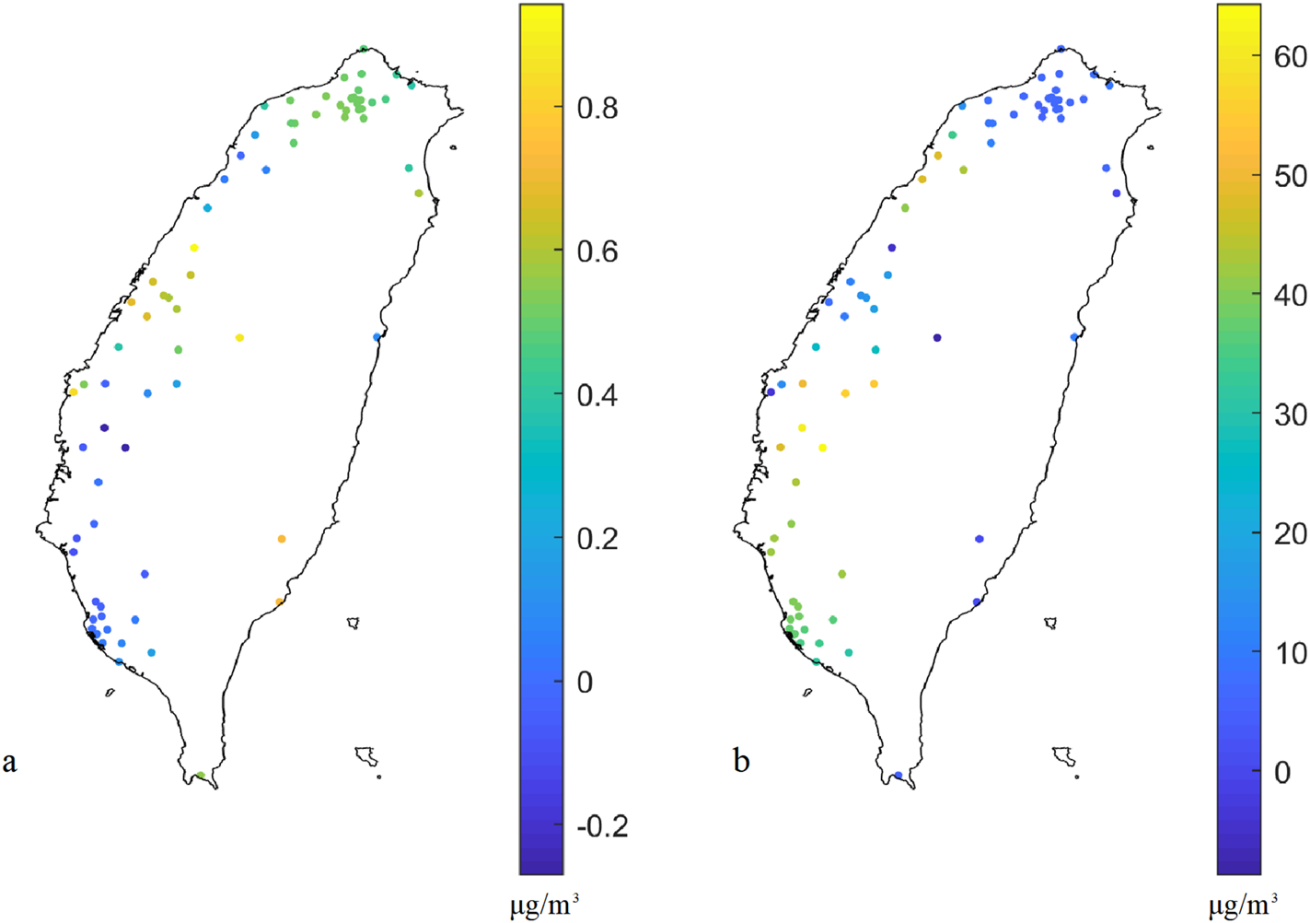
Coefficients of the spatial calibration model: (**a**) slope and (**b**) intercept based on the spatial calibration model (Eq. 4). The plots were created in MATLAB_R2018B (https://www.mathworks.com/products/matlab.html).

Figure 6 shows the spatial coefficients in the spatial calibration model and indicates that the coefficient varies with locations. When considering spatial calibration model, the average slope is 0.33, but the average intercept is 21.4 (the slope is 0.58 and the intercept is 9.4 in the nonspatial calibration model). The spatial calibration model can consider the effects of a spatially heterogenous pattern and explore spatial non-stationarity in the sensor calibration by allowing regression coefficients to vary spatially. Furthermore, the system in the sensor calibration is local linear with weights because sensor relationships exhibit spatially heterogeneous patterns.

### Model residual plots

**Figure 7:**
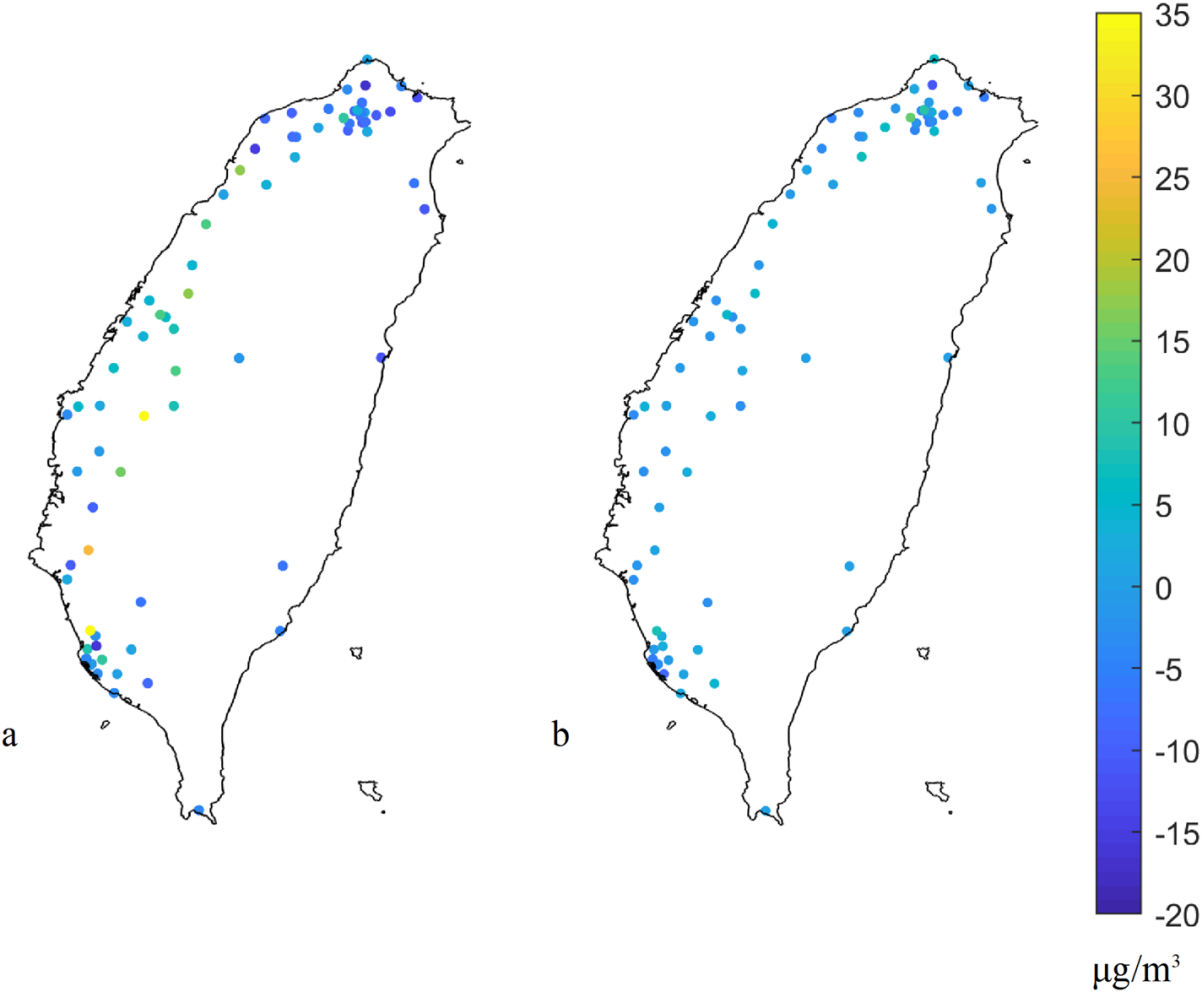
Patterns of model residuals from the (**a**) nonspatial and (**b**) spatial calibration models. The plots were created in MATLAB_R2018B (https://www.mathworks.com/products/matlab.html).

**Figure 8:**
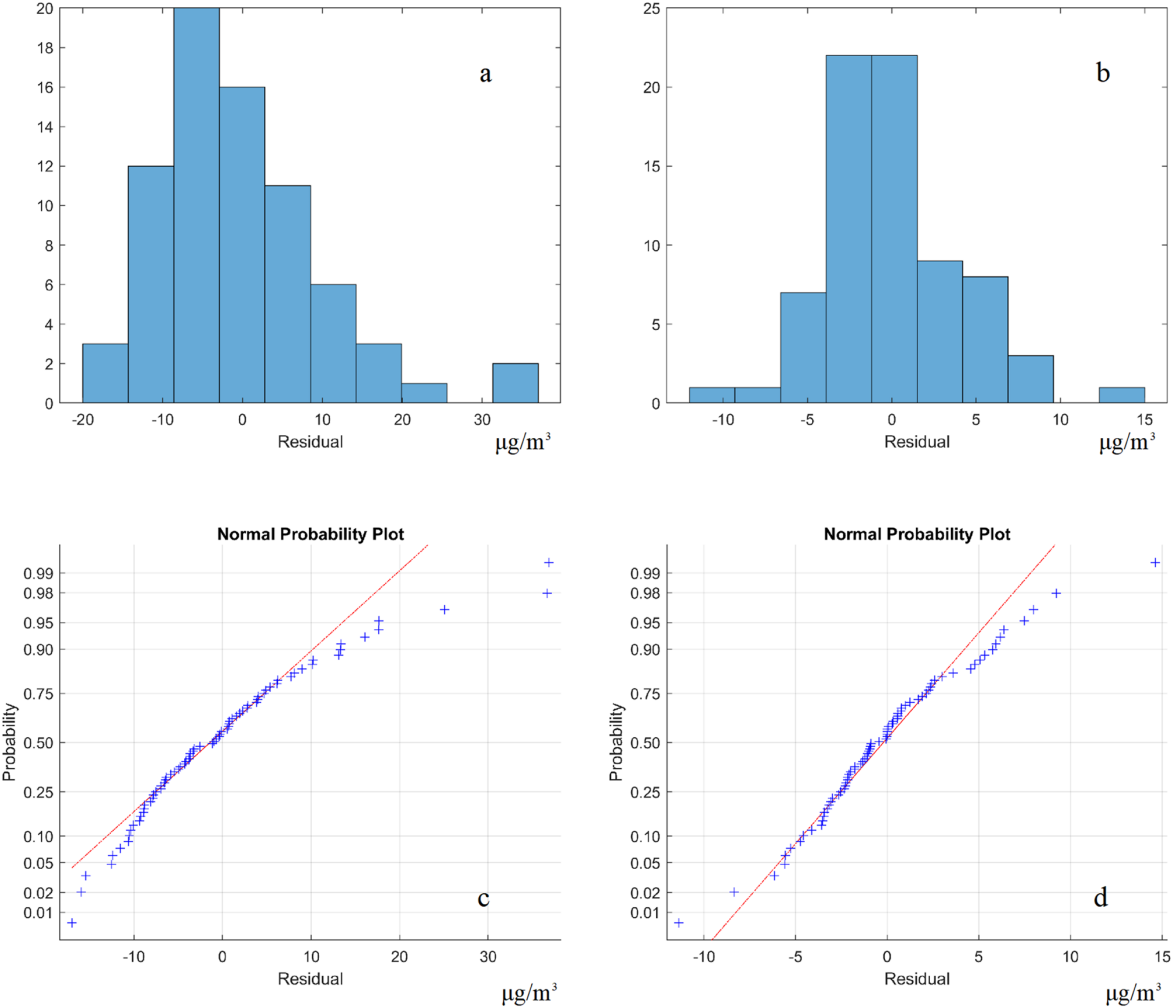
Residual histograms from the (**a**) nonspatial and (**b**) spatial calibration models, and normal probability plot of residuals from the (**c**) nonspatial and (**d**) spatial calibration models. The plots were created in MATLAB_R2018B (https://www.mathworks.com/products/matlab.html).

Examining residuals is a key part of statistical modeling. Figures 7 and 8 show the residual plots, residual histograms, and normal probability plot of residuals, respectively. The residuals of the nonspatial calibration model are between − 16 and 36 μg/m^3^, whereas the residuals of the spatial calibration model are between − 11 and 14 μg/m^3^. In Fig. 7, the overestimations of the PM_2.5_ concentration values exist in most areas of Taiwan, but the underestimations of the PM_2.5_ concentration values exist at the hotspots in the central and southern regions^21^. Hence, the spatial calibration model reduces the bias when compared to the nonspatial calibration model. The model, i.e., traditional linear regression, cannot work with the regional sensor data calibration, because the linear regression model is easily failed by spatial heterogeneity. After performing the calibration using spatial model, the results show the most random and normally distributed residual patterns in the spatial model (Fig. 8). The normal probability plot is a tool for assessing whether or not a data set is approximately normally distributed. The residual plot of the spatial calibration model shows a straight line, because the data set is approximately normally distributed. The smaller normally distributed residuals represent better model performance compared to the nonspatial methods.

### Spatial mapping of PM_2.5_

**Figure 9:**
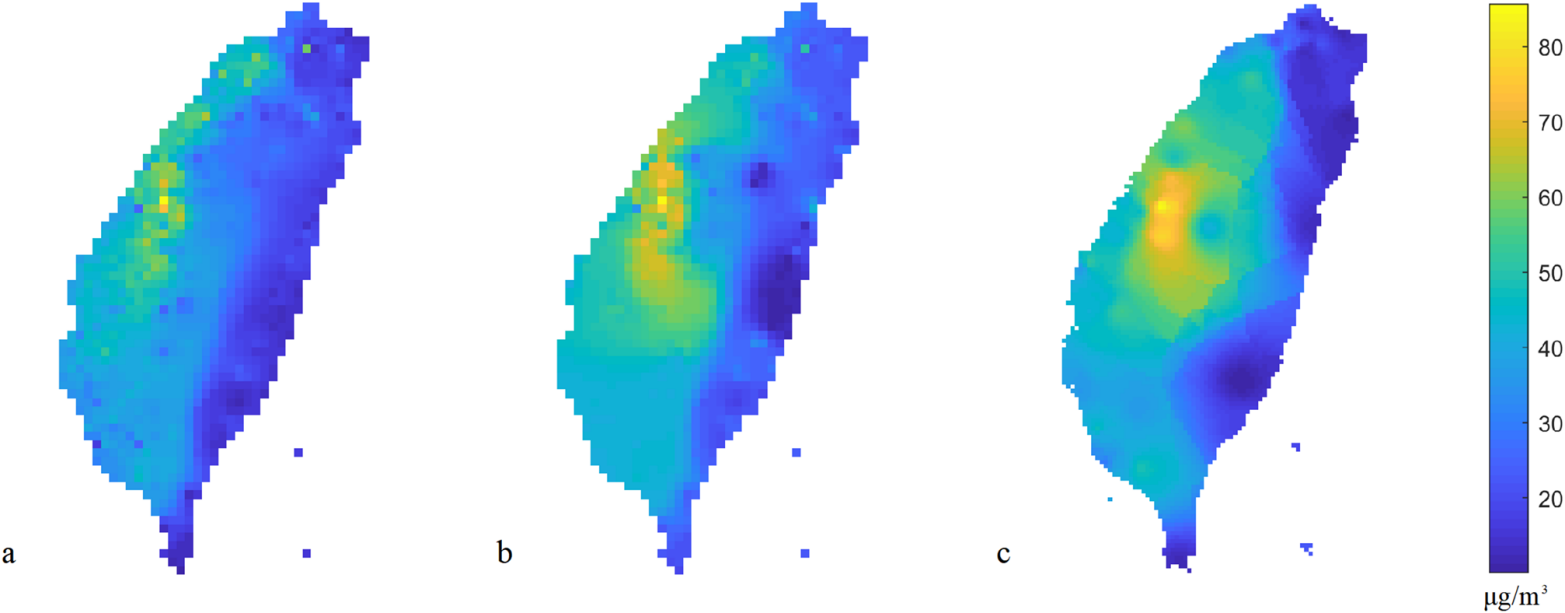
PM_2.5_ mapping based on calibrated low-cost sensors from (**a**) nonspatial calibration model, (**b**) spatial calibration model, and PM_2.5_ mapping based on (**c**) regulatory station data only. The plots were created in MATLAB_R2018B (https://www.mathworks.com/products/matlab.html).

In the nonspatial and spatial calibration models, the mapping RMSE values of PM_2.5_ concentrations are 7.9 and 4.8 μg/m^3^, respectively. The spatial calibration model can improve the bias and decrease the mapping of RMSE to 39% compared to the nonspatial calibration model. Figure 9 shows the PM_2.5_ estimation based on the nonspatial and spatial calibration models and regulatory stations. The hotspots from the low-cost sensors in the nonspatial calibration (Fig. 9a) are underestimated in the central and southwestern areas of Taiwan. After calibration, the spatial pattern of air pollution from low-cost sensors are similar to the results of the regulatory stations (Fig. 9b,c). The results (Fig. 9b,c) have similar large-scale patterns but vary in terms of details and spatial distributions in the local area. In addition, the spatial PM_2.5_ pattern from sparse regulatory stations are smooth and ideal. The spatial PM_2.5_ distribution from the low-cost sensors after calibration are complete and reasonable. Considering a large number of calibrated low-cost sensors, the spatial details can be shown in the estimation. The spatial pattern of air pollution showed that the air pollution levels of eastern and western Taiwan varied. The hotspots of air pollution are in the central and southwestern areas of Taiwan.

## Discussion

Strict collocation becomes a challenge when the low-cost sensor and regulatory networks are already established^2^. In this study, a nearest neighbor strategy was used by matching a low-cost sensor to its nearest TWEPA station. The overall average nearest neighbor distance of low-cost sensors is approximately 610 m, but the average nearest neighbor distance decreases to 311 m after excluding 7 data pairs with distances over 2 km. The high density of low-cost sensor network and the reasonability of this nearest neighbor strategy allowed for sufficient collocated samples to conduct the calibration.

Previous studies highlighted the association of the environmental conditions with the observation biases in low-cost sensors, as well as the calibration function that must be changed across space and time^7^. When used under environmental conditions with high relative humidity (> 80%), appropriate automated correction routines must be established to remove problematic observations from the datasets or transform these observations based on regulatory stations^1^. Increased relative humidity is associated with a near-exponentially increased low-cost sensor data bias^13^. The observed influences of high humidity on low-cost sensor bias may be related to the issues in electronic circuits and the hygroscopic growth of fine particulates^2^. In a time series, the PM_2.5_ bias and relative humidity show positive association, while the relative humidity is high, particularly over 85%^1,7^. In this case, 43% of sensors exist with the relative humidity over 85%, and the total of 56% sensors exist with the relative humidity over 80%. We easily faced this condition because the area had humid subtropical climate in the north and a tropical monsoon climate in the south of Taiwan. Therefore, low-cost sensors with high relative humidity must be established to eliminate the possibility of problematic observations. Multi-variable models may be fitted to obtain reasonable formulas, which can be easily interpreted and implemented from each sensor^22^. However, the current spatial calibration model performs the best (4.8 μg/m^3^), because the model is already considered a local weighting for the spatial heterogeneity of calibration. Not considering the environmental variables further may simplify the low-cost sensor data spatial calibration. When considering the relative humidity into the spatial calibration model, the model RMSE increases to 5.4 μg/m^3^. This result implies that the relative humidity shows a negligible association with the spatial calibration. The relative humidity is not the influencing factor in the spatial model, because the high relative humidity spreads everywhere at this time (average relative humidity is 82.1%).

Meanwhile, the calibration limitations and the risk of overfitting exist in small calibration datasets^23^. In practice, an appropriate and simple choice is to convert the low-cost sensor data to the reference system. The flexibility of an empirical spatial (location-specific) adjustment resulted in the consistent performance. After effective calibration, distributed low-cost sensors can help understand the spatial details of air pollution. In addition, data calibration commonly requires the space–time homogeneity of environmental conditions; however, the suitability of homogeneous assumption could not easily be validated^7^. If the environment is with high relative humidity, the real-time calibration for low-cost sensors is necessary. The calibration requires the real-time referenced and low-cost sensor data. The proposed approach did not find the general calibration equation, but needed to calibrate spatially at any time. In practice, the low-cost sensor will be real-time adjusted. In this case, computational time loading of the model is low (< 5 min) in the computer with Intel Core i5-10210U. The associations among low-cost sensors can be automatically converted to the reference system. In this case, the frequency of the monitoring systems of low-cost and regulatory sensors maybe inconsistent. The low-cost sensor updated rate is up to 5 min but the regulatory station is hourly updated. Thus, the real- or near real-time operation can only be set as the hourly calibration, then the real-time mapping of PM_2.5_ can be applied based on the current data with hourly-calibrated functions.

Low-cost sensors are cheap, compact, user-friendly, and provide high-resolution spatiotemporal air pollutant concentration data^3^. However, several types of low-cost sensors are also available for air quality monitoring in which the calibration may be applied diversely. In this spatial calibration model, the location-based and individual calibration coefficients at any time are considered.

This study offers an effective approach for exploring the low-cost sensor data transformation using the spatial calibration model, which is a local weighting method and is easy to fit between low-cost sensor and regulatory stations. The spatial calibration and mapping model provide additional details on the spatial variations of sensor transformation, which can be identified from the nonlinear and spatially varying relationship between the low-cost sensor and regulatory stations. Given that the observations of low-cost sensors play a critical role, the bias of the PM_2.5_ measurements from raw low-cost sensors can induce the biases of the risk perception from the public^7^. Even though the bias can be significantly improved through spatial calibration, the residuals can still be present in the result. For example, Fig. 9a,b show the differences in the observations from the low-cost sensors using nonspatial and spatial calibration. Such system biases were significantly reduced by the present calibration. Although the biases cannot be adjusted to exact zeros, the overall residuals were significantly mitigated in the spatial calibration process. Spatial calibration and mapping are very important in managing public perceptions of air quality levels. Using the spatial calibration, the spatial patterns of air pollution from low-cost sensors are consistent with those obtained from regulatory stations with high relative humidity. After spatial calibration, the pollution hotspots determined by the low-cost sensors can provide the detailed spatial patterns of PM_2.5_ better when compared to using regulatory stations. The pollution hotspots which were measured from the calibrated low-cost sensors happened in the central and southwestern areas of Taiwan.

## Conclusions

The study conducted spatial calibrations, which collected measurements of and investigated the spatial varying relations of low-cost sensors and regulatory stations. This work also presented the derivation of the spatial calibration approach for low-cost sensors given that the observed low-cost and regulatory measurements exhibited inconsistency and spatial heterogeneity.

The proposed model considered spatial regression and IDW interpolation for calibrating and mapping regional air quality. The low-cost sensor data can be adjusted using spatial calibration, especially in the environment with high relative humidity. Results showed that the proposed approach can explain the variability of the biases from the low-cost sensors with an R-square value of 0.94. The mapping RMSE values of PM_2.5_ concentrations decreased to 4.8 μg/m^3^. The spatial calibration model can reduce the bias and decrease the mapping of RMSE to 39% compared to nonspatial calibration. Moreover, the use of regional maps after spatial calibration—instead of regulatory stations—provided spatial information on air pollution. Spatial pattern of air pollution showed the reliable hotspots of air pollution in the central area and in the southwestern area of Taiwan. The proposed model provides planners with relevant information by which to understand air pollution after the spatial calibration of low-cost sensors based on regulatory observations. The spatial calibration and mapping model can effectively mitigate the misleading pollution risk perceptions from decreasing the biases of low-cost sensors in the environment with high relative humidity. In the future work, we will consider more spatial information, such as elevation into the kernel function. In addition, the model is available for both real-time calibration and offline calibration. The offline calibration will be applied based on long-term monitoring data.

Real-time visualization is available at https://sites.google.com/gs.ncku.edu.tw/ttest/%E9%A6%96%E9%A0%81
